# Computer-aided DSM-IV-diagnostics – acceptance, use and perceived usefulness in relation to users' learning styles

**DOI:** 10.1186/1472-6947-5-1

**Published:** 2005-01-07

**Authors:** Lars G Bergman, Uno GH Fors

**Affiliations:** 1 Dept. of Learning, Informatics, Management and Ethics (LIME), Karolinska Institutet, Berzelius way 3, S-171 77 Stockholm, Sweden

## Abstract

**Background:**

CDSS (computerized decision support system) for medical diagnostics have been studied for long. This study was undertaken to investigate how different preferences of Learning Styles (LS) of psychiatrists might affect *acceptance*, *use *and *perceived usefulness *of a CDSS for diagnostics in psychiatry.

**Methods:**

49 psychiatrists (specialists and non-specialists) from 3 different clinics volunteered to participate in this study and to use the CDSS to diagnose a paper-based case (based on a real patient). LS, attitudes to CDSS and complementary data were obtained via questionnaires and interviews. To facilitate the study, a special version of the CDSS was created, which automatically could log interaction details.

**Results:**

The LS preferences (according to Kolb) of the 49 physicians turned out as follows: 37% were Assimilating, 31% Converging, 27% Accommodating and 6% Diverging.

The CDSS under study seemed to favor psychiatrists with abstract conceptualization information perceiving mode (Assimilating and Converging learning styles).

A correlation between learning styles preferences and computer skill was found. Positive attitude to computer-aided diagnostics and learning styles preferences was also found to correlate.

Using the CDSS, the specialists produced only 1 correct diagnosis and the non-specialists 2 correct diagnoses (median values) as compared to the three predetermined correct diagnoses of the actual case. Only 10% had all three diagnoses correct, 41 % two correct, 47 % one correct and 2 % had no correct diagnose at all.

**Conclusion:**

Our results indicate that the use of CDSS does not guarantee correct diagnosis and that LS might influence the results. Future research should focus on the possibility to create systems open to individuals with different LS preferences and possibility to create CDSS adapted to the level of expertise of the user.

## Background

Different types of decision support (DS) methods have been used in medicine for long. Computerized decision support systems (CDSS) including so-called "expert systems" can be used in for example interpretation of medical images, medical diagnostics or other areas. Examples are MYCIN for antibiotic treatments [1] and deDombal's system for acute stomach pain [2]. In Psychiatry, the DIAGNO system by Spitzer and Endicott was described 1968 as a computer program that simulated a DSM-1 diagnosis based on data from the psychiatric status schedule [3]. The CATEGO and DETOX systems are other examples [4,5].

For a more general reading on CDSS adoption in medical practice, see Fieschi *et al *[6] and for different approaches used to implement computers as diagnostic aids in medical decision making see for example Engle Jr. [7] and Miller [8]. Another study has been published by Berner and colleagues [9] who compared performance scores between four different diagnostic decision support systems.

Various models, or mode of actions, of DS exists including textual guidelines based on if-then-else strategies that forces the decision maker to make decisions in a logical and sequential manner; more advanced systems using fuzzy logic; neural networks; and systems using so called Artificial Intelligence. Many DS systems have been developed with aims to enable more accurate, consistent diagnosis and faster diagnostic procedures. CDSS have been applied in many medical disciplines, but have also been discussed in terms of e.g. reliability, usefulness, and user-acceptance. For example, Lu *et al *[10] found that the willingness to use CDSS rely heavily on preferences and perceived usefulness. Another example is Ridderikhoff and van Herk [11] who stated that although physicians indicate a need for diagnostic support, medical diagnostic support systems are not in widespread use. Miller [8] pointed out that it is misleading regarding the state of the art of these systems to just focus on the lack of widespread use. Miller's bibliography of systems from 1954 to 1993 convinced him that diagnostic systems nowadays can be seen as ubiquitous and "The prospects for adoption of large-scale diagnostic systems are better now than ever before, due to enthusiasm for implementation of the electronic medical record in academic, commercial, and primary care settings." Friedman *et al *[12] indicated that CDSS should be able to improve healthcare quality by providing accurate, useful and timely diagnostic information to clinicians and that most studies have emphasized the accuracy of the computer system alone without placing clinicians in the role as direct users. In exploring the extent to which CDSS might improve the diagnostic capability of clinicians, the success rate varied between different groups with different training levels. Larger improvements were observed for students than for residents and faculty. They concluded that "hands on" use of CDSS might influence diagnostic reasoning of clinicians. Regarding the decision procedure of a real expert, there are a number of theories. Many of these points out that there are differences between the decision process of an expert and a beginner (novice) [13,14].

It is unclear if the perceived usefulness of CDSS only is due to the DS model itself, or if the design and management of the computerized system is as important. Furthermore, there have been discussions about the design of computer systems, and how they might suit different users with different user-characteristics. Allen [15] argues that individual differences between users of information systems might influence search performance. Different types of cognitive resources such as topic knowledge, search skills, cognitive abilities, cognitive styles and learning styles have been shown to be related to a variety of search tactics and to tendencies to use certain information system features [16].

### Learning styles

The concept of learning style (LS) might be regarded as an important characteristic and independent variable when individual differences in perceiving and processing information are investigated. A deep understanding of the user, his tasks and his environment is required to design a well-accepted (useful) computer program. Learning styles and its importance for users of computer systems has been demonstrated in various areas, for example in Internet use [17], web-based teaching [18], interactive multimedia environment [19], efficacy of computer training methods [20], hypertext environments [21], and in interactive learning systems [22].

There are a number of learning style models and inventories [23-27]. Claxton and Murrell [28] systematized the various learning style models based on Curry's [29] previous work on learning style constructs. The Kolb LS model [25], classified by Claxton and Murrell [28] as an information-processing model, has recently been further developed. The Kolb LS model has been widely applied during the years [30] and the latest version of the Kolb Learning Style Inventory instrument, is version 3 (LSI 3) [31,32].

**Figure 1:**
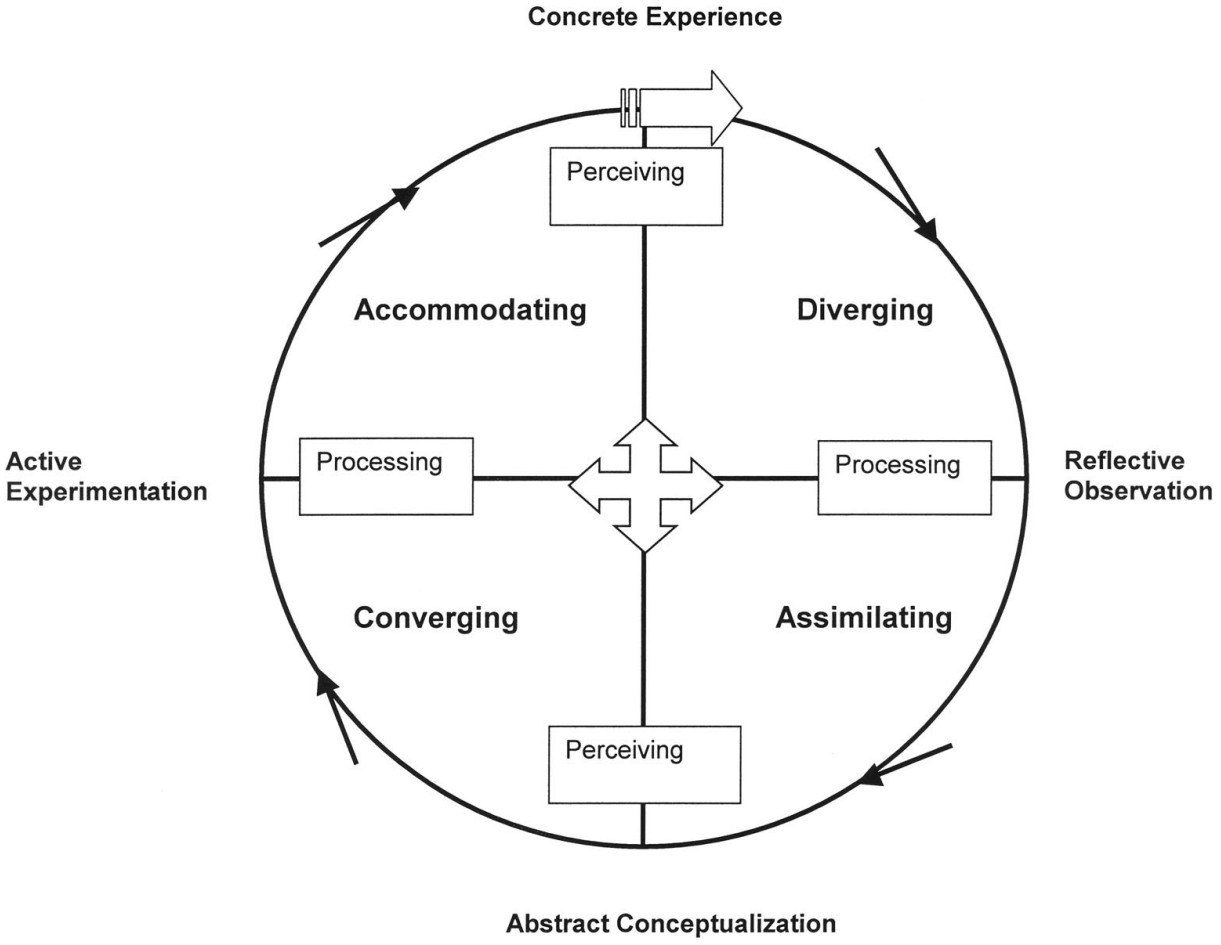
Structural Dimensions Underlying Learning Styles (After Kolb 1984)

This is a model of experience-based learning where all processes of the model are vital for the learning result. According to this model, the user/learner moves around the four modes in a circular direction (Figure 1). First there is an actual concrete learning experience. Second, the learner reflects on this experience. Third, the learner conceptualizes his/her observations and/or reflections into abstract theories or ideas. Fourth, the learner tests the theories or ideas by active experimentation. In this model, there are two processes for perceiving information: concrete experience mode and abstract conceptualization mode and two processes for processing experience into learning: active experimentation mode and reflective observation mode.

These four processes combine into four learning styles: Converging (abstract conceptualization mode and active experimentation mode), Accommodating (concrete experience mode and active experimentation mode), Diverging (concrete experience mode and reflective observation mode) and Assimilating (abstract conceptualization mode and reflective observation mode).

Diverging learning style is associated with value generating skills: building relationships, helping others and sense making (reasoning). Assimilating learning style is associated with thinking skills: information gathering, information analysis and theory building. Converging learning style is associated with decision skills: quantitative analysis, technical device use and formulation of goals. Accommodating learning style is associated with action skills: leadership, initiative and action [25,33]. Learning styles of the Kolb model are not only associated with skill, but also with adaptivity and flexibility concerning management of different situations. Curry [34] points out that a learning style is different from ability, strategy and tactic. Styles might be observed across content domains, abilities, personalities and interpersonal behaviors and they are measured in terms of typical performance. According to Curry, learning style is spontaneously demonstrated without conscious awareness or choice across a wide variety of situations with similar requirements. Strategies, in contrast are the result of conscious decisions and tactics are specific observable activities in specific performance situations [34].

### Diagnostics in psychiatry

In Psychiatry, the diagnostic process is mainly based on medical history, which is not often to be confirmed by lab tests or physical examinations. This leaves it more open for inter-personal differences in diagnostics, which might be a serious problem.

To facilitate the diagnostic procedures, and increase certainty and consistency in Psychiatry, a system called DSM has been created [35]. The DSM (Diagnostic Statistical Manual) system is a kind of information management system, an instrument that sort symptoms and handles them according to what the user judges as important symptoms. DSM is designed to assist the user by making the system criteria-based and by multi-axial descriptions, create a conflict-free base and thereby increase the reliability of the diagnosis. Currently, DSM version 4 (DSM-IV) is most used. The multi-axial DSM-system is based on the following reasoning:

1. Which symptoms have currently forced the patient to seek help?

2. How does the patient's overall pattern of experiences and behavior, compared with what is generally expected in the patients socio-cultural milieu, look like?

3. Are there any somatic diseases, which have to be attended to?

4. Have there occurred any stressful events in the patients' life along with the initial symptoms?

5. How serious are the problems just now, how is the patient functioning?

However, even DSM is not easily implemented in all situations, and therefore SCID, Structured Clinical Interview for DSM-diagnoses was created to further increase the reliability in psychiatric diagnostics [36]. SCID is a semi-structured interview instrument for DSM-IV-diagnoses, and is widely used in psychiatry internationally.

DSM training for physicians has been going on since 20 years during the Psychiatric course (9th semester) at Karolinska Institutet. The DSM-training is integrated in the course and the amount of time spent on DSM is approximately 4 hours. To our knowledge, there is no SCID-training in the Psychiatric course. However, in the training to become a specialist in Psychiatry at Karolinska Institutet, the DSM-framework is always used in teaching diagnostics and the amount of formal SCID-training is about 8 hours. Complementary to this, physicians becoming specialists in psychiatry are further trained in how to use SCID1 (axis 1 in DSM-IV) during their clinical training.

During a SCID interview, "jigsaw puzzle bits" are gathered, where DSM functions as a method to sort and put together these puzzle bits to known clinical syndromes. DSM is criteria based. For each criteria the users have to consider if the patient's symptom reach clinical significance so that the criteria can be regarded as fulfilled or not. There is some help for the interviewer in the DSM system in the form of a "decision tree" for axis 1 diagnosis.

### Computer support for SCID

CB-SCID1 (Computer-Based SCID for axis 1 diagnostics) is a software program that is reported to have advantages as compared to the ordinary SCID-interview (according to the CB-SCID1-manual) [37]. The program handles most administration tasks for e.g. summing up of fulfilled criteria and also presents an overview of noted diagnoses. The order of questions and some control of possible conflicting diagnoses are also taken care of by the program. The "decision tree" mentioned above is integrated in the software.

### Objectives

This study was undertaken to investigate and describe how different learning style preferences among psychiatrists might affect *acceptance*, *use *and *perceived usefulness *of the CDSS CB-SCID1 for DSM-IV-diagnostics.

## Methods

### Subjects

A number of practicing Psychiatrists, working at three different clinics, with different degree of expertise were asked to participate in this study. A fourth clinic was invited to participate in the study but could not do this due to lack of time. Out of 67 invited physicians, 49 volunteered to participate in the study. Out of these, 31 were experts at a senior level (being registered as specialists in psychiatry), and 18 were non-specialists (physicians with a position in psychiatry but without a specialist exam in psychiatry). In this study the groups are called "specialist group" (experts) and "non-specialist group" (not experts), respectively. They were all asked to complete a questionnaire regarding learning style preferences and to use the CB-SCID1 computer program for diagnostics of a real patient case (described in text) collected from the DSM-IV Case Book [38].

To be able to relate the Learning Styles of the 49 physicians in the study to the general situation in Sweden, a random sample of 250 (out of 1900 practicing psychiatrists in Sweden) were asked to fill in the same Learning Style inventories as mentioned above. This part of the study was done by sending out a letter including details of the study, which was followed up by a second letter as a reminder some weeks after the first one. All data were kept unidentified.

### Survey instrument for LS

The learning style preferences for all participating physicians were measured according to the Kolb model [25] using the Kolb Learning Style Inventory instrument, version 3 (LSI 3) [31,32]. This instrument (questionnaire) presents specific questions and statements, which the test person enters his personal views on. The responses entered are then used to categorize the LS preferences for the person under study [31,32].

### CDSS under study

The standardized terms and concepts of the DSM-system are the fundamentals of the CB-SCID1 system. CB-SCID1 uses logical inference of logical data (true, false), symbolically representing connections and dependency between components in the psychiatric knowledge base and presents questions according to the "paper" SCID-manual. CB-SCID1 takes care of the administration (for instance ordering of questions), correction possibility in criteria judgment, suggestion of answers according to previous in-data, summing up of fulfilled criteria, and also presentation of noted diagnoses. The system also handles some conflict control upon diagnoses. The user is asked to determine if various criteria are fulfilled or not and the system chooses how to go on, based on the user's input. If the number of fulfilled criteria reaches a certain level (according to DSM-IV) the program is automatically suggesting the corresponding DSM-IV diagnosis. The program is designed in a way that little training should be needed. CB-SCID1 also has a built-in, context sensitive help function in the consultation form, which put forward reminders and appropriate text information in tune with the decision problem at hand. The "assistance" from the system is based on the users input and also combined with the data driven rules derived from DSM-IV.

The physicians were instructed to use the CB-SCID-1 program as a tool to find the correct DSM-IV diagnoses of the paper case, and use the system as if the case had been a real one.

### Data collection

A special version of CB-SCID1, CB-SCID1_Log, was created, with a logging function that automatically stored a number of data in a separate log file, while the physician was using the program trying to make DSM-IV diagnosis on the patient case. The data logged (outcome log-file variables) were:

• Total session time (total time spent in the CB-SCID1-program)

• Total decision time (total time used to decide about the different criteria used in CB-SCID1)

• Average decision time (mean time to decide about a criterion)

• Total "non decision" time (time spent in the program not making decisions)

• Total number of criteria judged (total number of criteria decided about)

• Total number of diagnoses (total number (correct and incorrect), of proposed diagnoses by CB-SCID1)

• Total number of correct diagnoses (total number of correct diagnosis according to the DSM-IV Case Book)

• Total number of incorrect diagnoses (all other diagnoses proposed by CB-SCID1 and not correct according to the DSM-IV Case Book)

• Ratio between correct diagnoses and proposed number of diagnoses

• Total number of regretted criteria-judgments (total number of times the user clicks on the Regret-button in the CB-SCID1 while deciding about a criterion)

• Total number of criteria judged unclear (total number of times using the Unclear-button in the CB-SCID1 while deciding about a criterion)

• Sum of numbers of regretted criteria-judgments and unclear criteria judgments

The CB-SCID1_Log system (identical to CB-SCID1 for the user) was installed at the office computers of the clinicians and a short oral introduction of the system functionality was given, explaining both the use of the system, its online help system, and the aim of the study.

### Paper case used

The case used was picked from the DSM-IV Case Book [38]. The cases in this book are real, but unidentified, patients. These have been collected from a large number of clinicians (experts in particular areas of diagnosis and treatment). According to the Case Book, the recommended use of the cases is for example for researchers to assess the level of diagnostic expertise and the reliability with which members of their staff can make diagnostic assessments. A senior psychiatrist who is very experienced in DSM-IV and SCID training picked the actual case to be used. This specific case was chosen because it reflects multi-axial assessment (especially Axis 1 diagnoses), which was considered to be well suited for the CD-SCID1 program (which is aimed for Axis 1 diagnostics). The chosen case (called "Sickly" in the DSM-IV Case Book) is rather complex including three different diagnoses forcing the user to use the CB-SCID1 in full, taking decisions in problem areas like somatic problems, psychiatric problems and addiction problems.

The correct Axis 1 diagnoses (that is the assessment of Clinical Disorders and Other conditions that may be a focus of clinical attention) were in the actual case:

• Major Depressive Disorder, recurrent, mild

• Somatization Disorder

• Alcohol Dependence, in sustained full remission

To create a more realistic situation, the correct diagnoses were not revealed to the participating 49 psychiatrists, who all were given the same case with the aim to study the variation amongst the physicians using the CB-SCID1. They were not told what kind of case it was, neither the name of it ("Sickly"), nor where it came from.

### Investigation procedure

The investigation procedure was performed in four steps as follows:

1. *General information*: The project leader gave oral information at each clinic on a regular meeting for psychiatrists about the study

2. *Individual information and questionnaire*: Each participating physician was given further oral information about the study and was asked to fill in a form about gender, age, professional training, DSM/SCID training, computer skill and attitude towards computer-aided diagnostics. This was followed by filling in the Kolb Learning Style Inventory questionnaire LSI 3.

3. *CB-SCID1 test*: The physicians received oral instructions on how to use the computer program and that it was more or less self-instructive compared to normal "paper" SCID-training, before using CB-SCID1. They were then instructed to diagnose the patient case with the help of the CB-SCID1-system. Ten minutes were offered reading the patient case before starting the CDSS-system

4. *Follow-up interview and questionnaire*: A follow-up interview within a week from the first interview was done. There were open-ended questions about the pros and cons about the CB-SCID1 (pro and con categories were built on the basis of the answers content). Also structured questions using a four-graded scale were given about clinical interviewing skill (without computer aid), perceived usefulness of CB-SCID1 and computer anxiety, which all were graded using a four-graded scale.

### Statistical methods and analysis

Structured questions, constructed by the authors, and graded on a four-grade scale were given in the pre-assessment survey about computer skill and attitude to computer-aided diagnostics. In the post-assessment survey, structured questions were given about computer anxiety, clinical interviewing skill (without computer aid) and perceived usefulness of CB-SCID1. The questions were put in a clear statement which they could agree to/not agree to in an ordered categorical scale (Strongly disagree = 1, Disagree = 2, Agree = 3, Strongly agree = 4). The subject areas asked about were well defined and familiar to the users, why standardized attitude scales were not used. An open-ended question about the pros and cons about the CB-SCID1 were also given in the post-assessment survey. The answers to this question were grouped into several categories.

An analysis of correlations between the dimensions of LSI and the outcome log-file variables, as well as a comparison between specialists and non-specialists in this respect was also performed. Results were calculated as mean, standard deviation, median and lower – and upper quartile, where appropriate. Comparison between the two independent groups (specialists and non-specialists) was performed by the Mann-Whitney U Test and comparison between more than two independent groups (LS groups) was performed by the Kruskal-Wallis ANOVA by Ranks Test. Association between variables was calculated by Spearman Rank Order Correlations.

### Ethical approval

All parts of this study have been approved by the ethical committee of Karolinska Institutet.

## Results

### General results

All of the 49 psychiatrists volunteering to participate in the original study group fulfilled all phases of the study. The randomly sampled 250 psychiatrists had a response rate of 42% (95). Only 226 questionnaires could actually be sent out because 24 of the randomly chosen 250 psychiatrists could not be reached due to for example that they had moved abroad or retired. One of the 95 actual responses was incomplete. Demographic data of the study groups can be seen in Table 1.

**Table 1:** Demographic variables in study groups

**Variables**	**Original study group (n = 49)**	**Original study group – specialists (n = 31)**	**Original study group – non-specialists (n = 18)**	**Random sample group – specialists (Valid n = 93)**
Age (mean ± SD)	Male 48 ± 9 (n = 27)	Male 53 ± 7 (n = 14)	Male 43 ± 8 (n = 13)	Male 52 ± 8 (n = 46)
	Female 47 ± 11 (n = 22)	Female 52 ± 7 (n = 17)	Female 32 ± 6 (n = 5)	Female 50 ± 8 (n = 47)
				
DSM/SCID-training (hours, mean)	Male 7	Male 10	Male 3	Male 13
	Female 9	Female 11	Female 3	Female 10

The 49 physicians in the original study group were reporting a value of 3 for general computer skill and a value of 1 for computer anxiety, both median values on a four-graded scale (where 1 is very negative or very low and 4 is very positive or very high). There were no differences in computer skill between specialists and non-specialists. Computer skill median was 3 in the random sample group.

The physicians were reporting to have a good clinical interviewing skill (without computer-aid), the median was 4 for specialists and 3 for non-specialists, which would predict high expected values on correct diagnosis and low values on incorrect diagnoses.

In the original study group, the attitude to computer-aided diagnostics presented as medians and lower – and upper quartile were for, male specialists 3 (2–4), female specialists 3 (2–3) and male non-specialists 3 (3-3), female non-specialists 3 (3-3). In the random sample group, where all were specialists, the results were for males 3 (2–4) and females 3 (2–3).

Other general variables like Gender, Age, Level of professional training, Computer skill, Attitude to Computer-aided diagnostics, DSM-IV/SCID-training were not found to be statistically correlated to the dependent log file variables (Total session time, Total decision time, Average Decision Time, Total "non decision" time, Total Number of Criteria judged, Total Number of Diagnoses, Total Number of Correct Diagnoses, Total Number of Incorrect Diagnoses, Correct Diagnoses ratio, Total Number of regretted criteria-judgments, Total Number of Criteria judged Unclear and Sum of numbers of regretted criteria-judgments and unclear criteria judgments).

### Learning styles

The Learning Styles of the 49 physicians (tested by the LSI for learning style preferences) are shown in Table 2, where it is seen that the most common LS was Assimilating, followed by the Converging style. No major differences in Learning Style preferences were found between males (27) and females (22).

**Table 2:** Learning style preferences in the original study group and the random sample group

**Group**	**Assimilating**	**Accommodating**	**Converging**	**Diverging**	**Row Totals**
Original study group	18 (37 %)	13 (27 %)	15 (31 %)	3 (6 %)	49
*Specialists*	*10 (32 %)*	*6 (19 %)*	*12 (39 %)*	*3 (10 %)*	*31*
*Non-specialists*	*8 (44 %)*	*7 (39 %)*	*3 (17 %)*	*0 (0 %)*	*18*
Random sample group	34 (36 %)	23 (24 %)	17 (18 %)	21 (22 %)	95
**All groups**	52	36	32	24	144

In comparison, the random sample of the psychiatrists in Sweden (also tested by the LSI for learning style preferences) turned out as indicated in Table 2. Here was also the most common LS Assimilating, followed by Accommodating style. No significant differences were found among the genders.

Among the 49 psychiatrists, it was found a correlation (p < .01) between learning styles and reported computer skill. The persons with highest score on computer skill were Converging, followed by Accommodating and Assimilating. Diverging styles were found to have the lowest computer skill.

A positive attitude to computer-aided diagnostics and learning styles were also found to correlate (p = .04). Most positive were Assimilating, followed by Converging and Accommodating. Diverging had the lowest figures in terms of attitude to computer-aided diagnostics.

Finally, it was also found that the distribution of learning styles and the Number of Criteria judged in the system significantly correlated (p < .01). The Accommodating group used the highest number of criteria, followed by the Converging, Diverging and Assimilating groups in that order.

### Diagnostic results

Interestingly, the 49 physicians gave a rather low percentage of correct diagnoses. The correct diagnoses Major Depressive Disorder was found by only 27%, Somatization Disorder by 55% and Alcohol Dependence by 78%. Only 10% had all three diagnoses correct. 41% two correct diagnoses, 47% one correct diagnose and 2% no correct diagnose at all. Median values of proposed, correct, incorrect and ratio correct/proposed diagnoses for specialist and non-specialists are shown in Table 3.

**Table 3:** Median values of proposed, correct, incorrect and ratio correct/proposed diagnoses

**Group**	**Proposed diagnoses**	**Correct diagnoses**	**Incorrect diagnoses**	**Ratio correct/proposed**
Original study group (n = 49)	4	2	3	40 %
*Specialists (n = 31)*	*4*	*1*	*3*	*33%*
*Non-specialists (n = 18)*	*4*	*2*	*3*	*50 %*

As seen in Table 3, the non-specialist group seems to produce slightly better results (although not significant) than the specialist group. Also visible in the table, the median values for the number of proposed diagnoses (as a result from CB-SCID1 use) was as high as 4 for both specialists and non-specialists compared to the three correct diagnoses, which makes the above findings even more interesting and indicates a risk of over-diagnosing.

### Acceptance

The indicator of acceptance, user's attitude to computer-aided diagnostics, was found to be 3 on a four-graded scale for both specialists and non-specialists in the original study group as well as for the random sample. No difference was found among genders. When correlating this to computer skill and computer anxiety it was found an overall negative significant correlation (-.48, p < .001) between positive attitude to computer-aided diagnostics and computer anxiety. Within the Accommodating group there was an even higher negative significant correlation (-.80, p < .001) between positive attitude to computer-aided diagnostics and computer anxiety.

There was only a tendency to a correlation between computer skill and a positive attitude toward computer-aided diagnostics (.27, p = .06) in the original study group. However, within the Accommodating group, there was a positive significant correlation (.62, p = .02). In the random sample group, a significant correlation between computer skill and positive attitude to computer-aided diagnostics (.43, p < .001) was found.

Finally, there was an overall negative significant correlation between computer skill and computer anxiety (-.51, p < .001).

### Use

The use of CB-SCID1 was found to be varying among the 49 psychiatrists. For example, a positive significant correlation (.31, p = .03) was found between the active experimentation information processing mode (Converging and Accommodating) and the Total Number of Criteria judged in the program. No other significant correlations between the active experimentation-reflective observation dimension and the other 11 outcome log-file variables were found.

The total number of criteria judged for the patient case used varied from 44 to 119.

There were no significant correlations between the abstract conceptualization-concrete experience dimension and the 12 outcome log-file variables.

The comparison between the specialist group and non-specialist group concerning the 12 log-file variables revealed no significant differences of medians (See Table 4).

**Table 4:** Comparison of medians, lower – and upper quartile between specialists and non-specialists in outcome log-file variables

**Variable**	**Original study group (n = 49)**	**Specialist group (n = 31)**	**Non-specialist group (n = 18)**	**p-value**
Tot time (seconds)	1895 (1476–2405)	1710 (1476–2132)	2064 (1457–2532)	0.32
Decision time (seconds)	1193 (870–1559)	1035 (844–1486)	1269 (1107–1758)	0.20
Average decision time (seconds)	17 (13–22)	15 (12–20)	19.5 (14–22)	0.18
"Non decision time" (seconds)	661 (495–800)	650 (495–772)	677 (454–900)	0.83
Criteria judged (number)	68 (61–78)	69 (61–78)	67.5 (61–78)	0.74
Proposed diagnoses (number)	4 (3–5)	4 (3–5)	4 (4–5)	0.88
Correct diagnoses (number)	2 (1–2)	1 (1–2)	2 (1–2)	0.50
Incorrect diagnoses (number)	3 (2–4)	3 (2–4)	2.5 (2–3)	0.57
Ratio correct/prop diagnoses	40 (25–50)	33.3 (25–50)	50 (25–50)	0.24
Regretted judgments (number)	3 (1–8)	2 (1–8)	3.5 (2–10)	0.32
Unclear judgments (number)	7 (3–15)	7 (3–15)	8.5 (3–15)	0.80
Sum regretted unclear judgments (number)	14 (8–23)	14 (6–22)	13 (8–29)	0.56

### Perceived usefulness

When analyzing the follow-up interview, (after trying CB-SCID1) a significant correlation (-.32) between perceived future usefulness and the Abstract Conceptualization – Concrete Experience dimension (p = .02) was found. The Abstract Conceptualizations orientated group had more Pros while the Concrete Experience orientated had more Cons on perceived future usefulness. This indicates that the Assimilating and Converging learning styles, which perceive information by abstract conceptualizations, are favored by the CB-SCID1-system.

According to the interviews, the perceived usefulness of the CB-SCID1-system was more negative than positive. 27 (55%) of the psychiatrists noted more Cons than Pros, 22 noted more Pros than Cons (45%).

The responses were also categorized according to their general content. 6 positive and 7 more negative general categories were found, see Table 5.

**Table 5:** Pro and Con categories of Perceived usefulness of CB-SCID1

**Pros **(6 different categories)	**Cons **(7 different categories)
• **Structure **"there is a structure to hold on to in the program"	• **Appropriateness **"not suited for the diagnostic interview situation"
• **Accurate and reliable diagnoses **"contradicts diagnoses by feeling", "more exact diagnose"	• **Empathy and intuition **"risk of missing emotional and non-verbal information"
• **Feedback **"what works in treatment or not"	• **Conflict **"managing the patient contact and the CB-SCID1-system at the same time"
• **Help **"help in diagnostic thinking while working with the program"	• **Underestimation **"risk of underestimating your own skill, risk of getting dependent of the program"
• **Correction **"you will be noticed if you are on the wrong track"	• **Routine questioning **"promotes exhaustion effects and lack of initiative"
• **Timesaving **"the program runs all administration and presents the diagnostic results"	• **Dialogue **"breaking up of dialogue, missing emotional states and risk of irrelevant questions"
	• **General picture **"risk of losing the overall picture"

## Discussion

### General results

Given the reported high clinical interviewing skill, high computer skill and relatively positive attitude to computer-aided diagnostics for the group, the low number of correct diagnoses and high number of incorrect diagnoses is very interesting. The DSM-IV-diagnosis is a symptom-diagnose derived from a deliberately limited amount of relevant diagnostic information, pattern of symptoms and development within predefined limits. This is not intended to be compared with the clinical diagnosis, which is building on patterns of symptoms, complete development, actual circumstances, anamnestic data, etiological discussion, laboratory tests, psychological tests etc. The symptom-diagnose and clinical diagnose perspective might be complementary to each other and it is usually recommended that a clinical interview always should be done before the SCID or CB-SCID1 interview. One possible explanation of our findings is that diagnosis in psychiatry is so complex that neither the DSM-system nor the "paper-SCID" or the CB-SCID1 systems might help. Another possible explanation is that CDSS have their limitations. This is in accordance with for example Dreyfus and Dreyfus who argued that computers could be good competent manipulators of symbols according to prepackaged algorithms, but they lack the type of intuition that real experts have [39].

There are alternatives to the SCID and CB-SCID1 to make DSM-IV-diagnosis. For example, the SCAN (Schedules for Clinical Assessment in Neuropsychiatry) and the computerized version, the SCAN 2.1 system, are systems developed by the WHO (World Health Organization) [40,41]. The computerized SCAN system is more based on "facts" from the patients' answers transformed by algorithms to DSM-IV diagnosis compared to the CB-SCID1 where the interviewer's judgment of each criterion is of importance. If a system like SCAN would have given other results, remains to be investigated.

The fact that this study did not diagnose living patients and that it was done with the help of a computer program may affect the results in that tacit knowing could not come into play as much as in a real situation. Polanyi [42] mentions that we can notice and do things without being able to tell how we recognize something or tell exactly what we do. Maybe the results of the experts, number of correct diagnoses for instance, are most affected by this in their intuitive way of functioning due to extensive personal experience, exercise and experience of former master-trainee relationship that is, "tacit knowing knowledge".

We also have to keep in mind that the 49 physicians tried the CB-SCID1 for the first time and with limited training in the program (even if the system is said to be possible to be used with little training). When trying to evaluate the results, we have to consider the different needs, habits and working style of experts and non-experts of different levels. For example, any expert, with a possible intuitive way of thinking, might be confused when they use a program addressed to non-experts that emphasize rule-following and logical step-by-step working procedures.

But our interpretation is still that the explanation is that CDSS do not suit all clinicians. This is in accordance with for example Ridderikhoff and van Herk [11] who found that despite need for diagnostic help, computer-aided support systems was ranked lower than other computer-aids and the use of a diagnostic computer-aided support system.

### Limitations of the study

A limitation of this study is that only one case was used. The main reason for only using one single case was that the 49 physicians participating were very busy and reported that they had little time to spend for research projects like this. Furthermore, most of the 18 physicians not accepting to participate in the study reported lack of time as the main reason; asking the participants to use more cases, would most probably have resulted in more dropouts. The fact that physicians decided not to participate due to time constraints, could also raise a concern about selection bias since they might actually be less prone to use computer technology, differ in learning style or else. However, as the real case used is judged as being rather standardized and has been used successfully in training, we consider the risk of incorrect results being rather low.

Another limitation is that the computer skill was self-reported, and that no standardized skill test was used.

Finally, one limitation is that some of the psychiatrists participating in this study might have seen the used case before. However, since the DSM Case Book covers more than 200 cases, the chance that those psychiatrists remembered both the actual case and its three specific diagnoses is considered to be very low.

### LS and psychiatry

It has been reported that domain specialists might have different LS preferences, for example Baker III, *et al *[43] using the Kolb model found that there is an identifiable surgical learning style: Converging (46 %). The other styles were Accommodating (26%), Assimilating (20%) and Diverging (8 %). This is in line with the Plovnick [44,45] results, which suggests surgeons as Converging in Medicine. Our results concerning learning styles did not confirm the Plovnick results that psychiatrists should be Diverging. This could depend on various reasons. The role for the psychiatrist today is not the same as it was 30 years ago. The "Diverging" aspect of relational skill is less vital compared to diagnostic methods of today and various modern treatment methods.

Another possible explanation is, according to the Kolb theory, Diverging-preference persons with their Concrete perceiving and Reflective observation are predicted to be the least interested in computer work, and thus that there is a possibility that the result is due to selection bias and low response rate. Theoretically the nearly 60 % of the 226 Swedish psychiatrists who did not answer the questionnaire, might have a Diverging preference. If so psychiatrists would have a Diverging preference, and the CB-SCID1 perceived usefulness values would probably be even more negative. Divergers also had the lowest figures in terms of attitude to computer-aided diagnostics and computer skill. Could it be that the low number of Diverging preferences, 6% in the original study group is the result of "selection bias"? After all, they were volunteers to participate in the study. They were asked to try the CB-SCID1 and may have hesitated to participate due to lack of computer skill? The random sample group was not asked to try the CB-SCID1 and Diverging preferences in that group were 22%. The higher numbers with Converging preference (high computer skill) in the Original study group compared to the Random sample group might have been explained by the same reason.

The fact that there were no significant correlations between the general variables and the dependent log-file variables indicate that further analyses of for example attitudes, use, perceived usefulness and learning style must be made in the future. This is supported by our findings that individual values regarding computer skill, computer anxiety and attitude to computer-aided diagnostics are related to learning styles and acceptance, use and perceived usefulness.

### Acceptance

Computer skill, computer anxiety and the attitude to computer-aided diagnostics are interpreted to be important variables in the acceptance of the CB-SCID1-system. Accommodating psychiatrists with computer anxiety have a very low value on the indicator of acceptance. This means that this group has difficulties accepting the CB-SCID1. Again within the Accommodating group, those with computer skill are positive to the indicator of acceptance.

This highlights the importance of training as a means to increase computer skill and ease the acceptance of systems like CB-SCID1. Maybe the acceptance would increase if only targeting beginners or persons on "medium level" of training. As shown, a tendency for a more positive regard to computer-aided diagnostics exists in the non-expert group. The specialists, as proficient and on expert level, might not have a need of such computer-aid, or at least a different need in accordance with their way of working as experts with an intuitive frame of reference compared to the step by step, rule-following work of the novice.

### Use

A possible explanation for the low number of significant results between LS and the outcome log-file variables might be that learning style is used with more flexibility in a real situation (using CB-SCID1) compared to more "attitude-like" variables like acceptance and perceived usefulness.

It might be so that LS must be linked to learning skill and adaptivity/flexibility in different specific situations to give significant results against the log-file variables. The LSI is measuring learning style preferences not associated to specific situations.

### Perceived usefulness

Our findings that the program seems to be more attractive to psychiatrists with learning styles which prefer Abstract Conceptualizations (Assimilating and Converging learning styles) and in the majority negative to the Concrete Experience (Accommodating and Diverging learning styles) is in accordance with Lu *et al *[10] who found that the willingness to use CDSS rely heavily on preferences and perceived usefulness.

The Cons in future perceived usefulness against the CB-SCID1 about the appropriateness of such a program, the conflict perspective about using it and the breaking up of dialogue contain an all or none view. However, the Cons arguments have much in similar with the Dreyfus and Dreyfus standpoint regarding the limitations of computer use and seems to be reflecting the expert view, for instance lack of appropriateness, empathy and intuition and underestimation of your own skill. The Pros arguments seem to meet the non-expert view of learning to diagnose, for instance the program as a structure, help and correction facility. How to use, by whom and when, in which situations the CB-SCID1 is to be used must be elaborated upon. Non-experts with Diverging preferences might increase their flexibility using learning styles as a result of training and maybe a more positive regard to computer-aided diagnostics. That is, going around the "Kolb circle" in a more flexible and balanced way, using all learning styles, and give up their strong Diverging preferences.

### Overall results from acceptance, use and perceived usefulness

Our results indicate that computer skill is of importance, and computer anxiety of negative impact on the attitude to computer-aided diagnostics. The highest computer skills were found within the Converging and Accommodating groups, which use Active Experimentation as information processing mode. Most positive to computer-aided diagnostics were the Assimilating and Converging groups, which in perceiving information are using Abstract Conceptualization.

The results also indicate that the Active Experimentation information-processing mode (Accommodating and Converging learning styles) is significantly correlated to number of criteria judged in the program.

Furthermore, the results also indicates, that although the 49 psychiatrists reported a positive attitude to computer-aided diagnostics, physicians with computer anxiety are less positive. Moreover, the CB-SCID1 CDSS seemingly invites the Accommodating and Converging learning styles to significantly adopt an Active Experimentation information-processing mode with a high number of criteria judged. However, this experimental mode may increase the number of incorrect diagnoses. The Assimilating and Converging learning styles also seems to be favored by the computer program concerning the Abstract Conceptualization mode in perceiving information.

## Conclusions

The results of this study suggests that a CDSS is no guarantee of improved diagnostic procedures in Psychiatry and that even a clinically experienced user might end up with several incorrect diagnoses using such a system. The results also indicate that the use of CDSS-tools seems to favor users with learning style preferences using abstract conceptualization information perceiving mode.

Furthermore, our results indicate that future research on CDSS should focus on the possibility to create systems open to individuals with different LS preferences. Future research should also focus on the importance of computer training and different professional levels to optimize the usefulness of CDSS. The relationship between learning style preferences and working style habits at different professional levels might also be elaborated upon as well as the importance of learning style flexibility and decision modes in various diagnostic situations.

## List of abbreviations used

CDSS Computerized decision support system

DS Decision support

DSM Diagnostic Statistical Manual, "DSM-system"

DSM-IV Diagnostic Statistical Manual, version 4

LS Learning Style

SCID Structured Clinical Interview for DSM-diagnoses

CB-SCID1 Computer-Based Structured Clinical Interview for DSM-diagnoses (axis 1)

## Competing interests

Competing interests exists between the commercial CB-SCID1 system from Pilgrim Press and the SCAN-system developed by WHO, World Health Organization. However, the authors have no interests in any of these competitors.

## Authors' contributions

UF has supervised LB in the planning, carrying out and analysis of this research work. LB has carried out all experiments. The manuscript has been written on close collaboration between LB and UF.

## Pre-publication history

The pre-publication history for this paper can be accessed here:


